# c-Myc deregulation induces mRNA capping enzyme dependency

**DOI:** 10.18632/oncotarget.12701

**Published:** 2016-10-16

**Authors:** Olivia Lombardi, Dhaval Varshney, Nicola M. Phillips, Victoria H. Cowling

**Affiliations:** ^1^ Centre for Gene Regulation and Expression, School of Life Sciences, University of Dundee, Dundee DD1 5EH, UK; ^2^ School of Science and the Environment, Manchester Metropolitan University, Manchester, M15 6BH, UK

**Keywords:** c-Myc, mRNA cap, transcription, translation, cell proliferation

## Abstract

c-Myc is a potent driver of many human cancers. Since strategies for directly targeting c-Myc protein have had limited success, upstream regulators and downstream effectors of c-Myc are being investigated as alternatives for therapeutic intervention. c-Myc regulates transcription and formation of the mRNA cap, which is important for transcript maturation and translation. However, the direct mechanism by which c-Myc upregulates mRNA capping is unclear. mRNA cap formation initiates with the linkage of inverted guanosine via a triphosphate bridge to the first transcribed nucleotide, catalysed by mRNA capping enzyme (CE/RNGTT). Here we report that c-Myc increases the recruitment of catalytically active CE to RNA polymerase II and to its target genes. c-Myc-induced target gene expression, cell proliferation and cell transformation is highly dependent on CE, but only when c-Myc is deregulated. Cells retaining normal control of c-Myc expression are insensitive to repression of CE. c-Myc expression is also dependent on CE. Therefore, inhibiting CE provides an attractive route for selective therapeutic targeting of cancer cells which have acquired deregulated c-Myc.

## INTRODUCTION

*c-myc* is an essential gene required for cell growth and proliferation [1, 2]. In response to growth factor signalling c-Myc is upregulated and promotes the expression of genes which drive cell proliferation. Under homeostatic conditions the *c-myc* gene is tightly regulated. However, *c-myc* is deregulated in over half of human cancers, which directly contributes to oncogenic transformation [3, 4].

c-Myc is a basic helix-loop-helix leucine zipper (bHLH-LZ) transcription factor which forms a heterodimer with Max, another bHLH-LZ protein. In this complex, c-Myc binds to E-box sequences proximal to transcription initiation sites and regulates the transcription of target genes [5, 6]. c-Myc increases transcription by recruiting histone acetyltransferases and RNA polymerase II (RNA pol II) kinases [7–11]. In addition, a subset of genes is suppressed by c-Myc using various mechanisms, including displacement or inhibition of other transcriptional regulators, recruitment of histone deacetylases and activation of Ezh2 methyltransferase [2, 12, 13].

Global analyses revealed that c-Myc regulates transcription of 10-15% of all genes [2, 14, 15]. The genes regulated by c-Myc vary between cell types with the exception of a core signature of genes which promote cell growth, including those involved in ribosomal biogenesis, nucleolar function and RNA processing [16, 17]. c-Myc also directly promotes RNA polymerase I and III-dependent transcription [18]. When expressed at oncogenic levels, c-Myc binding specificity becomes less stringent and it regulates all actively transcribed genes, although less so than its canonical targets [19, 20]. The model emerges that c-Myc deregulation directly regulates a subset of genes and causes amplification of general transcription and translation, ultimately increasing the propensity of cells to undergo oncogenic transformation [16, 19, 21].

c-Myc-dependent RNA pol II phosphorylation has the potential to regulate recruitment of factors important for transcription and mRNA processing to its targets genes [7, 8, 22–25]. Factors that promote transcription initiation, elongation, mRNA capping, splicing and transcription termination are recruited to the phosphorylated C-terminal domain of RNA pol II. c-Myc regulates formation of the mRNA cap on its target transcripts, which contributes to c-Myc-dependent gene expression and cell proliferation [8, 26, 27]. The mRNA cap is 7-methylguanosine linked to the first transcribed nucleotide, a structure which protects transcripts from degradation, promotes splicing and 3′ end processing, facilitates nuclear export of mRNA, and aids loading of mRNAs onto ribosomes for translation [28].

The direct mechanism by which c-Myc promotes mRNA capping is unclear. The enzyme which initiates cap formation, mRNA capping enzyme (CE/RNGTT), specifically binds to the RNA pol II CTD when phosphorylated on Ser-5, spatially and temporally localising the enzyme to act on nascent transcripts [29–34]. CE has triphosphatase and guanylyltransferase activites which act sequentially to add the guanosine cap structure to transcripts. CE triphosphatase cleaves the terminal phosphate from the first transcribed nucleotide and CE guanylyltransferase transfers guanosine mono-phosphate (GMP) to the transcript 5′ end, creating the guanosine-capped structure. Ser-5 phosphorylated RNA pol II CTD also activates CE guanylyltransferase activity *in vitro* [35]. The final step in basic cap formation is catalysed by RNA guanine-7 methyltransferase (RNMT), which methylates the guanosine moiety at the N7 position. RNMT is also recruited to transcription initiation sites, probably via the phosphorylated RNA pol II CTD, since recruitment is diminished by CTD kinase inhibitors [30, 36]. Although CE and RNMT are recruited to phosphorylated RNA pol II, to date, their recruitment has not been demonstrated to be c-Myc-dependent. c-Myc-dependent cap methylation requires upregulation of the c-Myc target gene S-adenosyl homocysteine hydrolase, (SAHH) [27]. SAHH hydrolyses the inhibitory by-product of methylation reactions, SAH, thus enhancing mRNA cap methylation. However, increased expression of SAHH alone is not able to elevate mRNA cap formation and therefore c-Myc-dependent SAHH upregulation permits rather than promotes cap formation.

We investigated the relationship between c-Myc and CE. We found that c-Myc increases the recruitment of CE to RNA pol II and to proximal promoter regions. Deregulated c-Myc is highly dependent on CE to drive gene expression and cell proliferation, whereas endogenous c-Myc is not. We also report that the expression of deregulated and endogenous c-Myc is dependent on CE.

## RESULTS

### c-Myc deregulation increases the interaction of RNA pol II with CE

**Figure 1:**
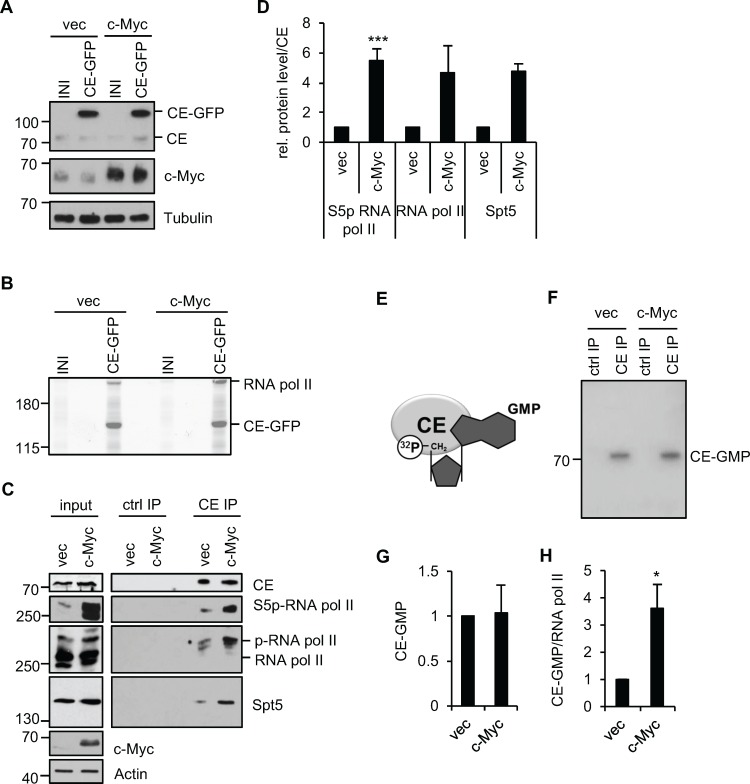
c-Myc increases the interaction of CE with RNA pol II (**A**) Protein extracts from IMEC/vec and IMEC/c-Myc, expressing CE-GFP or empty vector (INI) were analysed by Western blotting. (**B**) CE-GFP complexes were immunoprecipitated, resolved by SDS-PAGE and Coomassie-stained. Mass spectrometry was used to identify proteins. (**C**) CE complexes were immunoprecipitated from IMEC/vec and IMEC/c-Myc and analysed by Western blotting. FLAG antibody used as a control (ctrl IP). Input and immunoprecipitation (IP) panels are different exposures of the same Western blots. (**D**) ImageJ software was used to quantify Western blot signal for S5p-RNA pol II, RNA pol II pan and Spt5 in CE IP, normalised to CE. Error bars represent standard error of the mean, n=4 (S5p-RNA pol II) or n=2 (RNA Pol II and Spt5). (**E**) CE hydrolyses [α-^32^P] GTP, yielding a ^32^P-labelled CE-GMP intermediate, an approximation of CE activity. (**F**) CE IPs from nuclear extracts of IMEC/vec and IMEC/c-Myc were incubated with [α-^32^P]GTP, resolved by SDS-PAGE and ^32^P detected by phosphorimager. (**G**) Signal quantified by phosphoimager. Error bars represent standard error of the mean, n=5. (H) RNA pol II was immunoprecipitated from IMEC/vec and IMEC/c-Myc and ^32^P-GMP binding detected by phosphoimager. CE-^32^P-GMP signal normalised to RNA pol II (detected by Western blot). Error bars represent standard error of the mean, n=4. Significance relative to control calculated by Student's T-test; * = p≤0.05, *** = p≤0.001.

To investigate the relationship between c-Myc and mRNA capping, we determined the impact of *c-myc* deregulation on the proteins with which capping enzyme (CE) interacts. This analysis was performed in human immortalised mammary epithelial cells (IMECs), which are non-transformed and exhibit many normal growth controls [37]. Deregulation of c-Myc transforms IMECs, resulting in elevated proliferation rate and anchorage-independent growth [38]. Retroviral infection was used to create IMEC lines which express elevated levels of c-Myc or vector control, designated IMEC/c-Myc or IMEC/vec, respectively. A second round of infection resulted in stable expression of CE-GFP (C-terminal GFP fusion) or the empty vector control (INI). Equivalent levels of CE-GFP were expressed in IMEC/c-Myc and IMEC/vec and CE-GFP expression did not alter c-Myc expression (Figure 1A). CE-GFP complexes were immunoprecipitated from IMEC lines via the GFP tag and resolved by SDS-PAGE. Mass spectrometry was used to identify CE-GFP-interacting proteins, which included RNA polymerase II large subunit (RNA pol II), a previously observed CE-interacting protein (Figure 1B) [31, 33, 39]. Increased c-Myc expression resulted in more RNA pol II being purified with CE-GFP (Figure 1B). To confirm this observation, endogenous CE complexes were immunoprecipitated from IMEC/c-Myc and IMEC/vec (Figure 1C and 1D). As observed previously, deregulation of c-Myc resulted in increased RNA pol II C-terminal domain (CTD) Ser-5 phosphorylation [8]. Deregulated c-Myc expression resulted in increased interaction of CE with phospho-Ser 5 RNA pol II, observed using phospho-specific and pan RNA pol II antibodies. Previous studies reported that Spt5, a mediator of RNA pol II elongation, binds to RNA pol II and CE [40–42]. Elevated c-Myc expression also increased the interaction of CE with Spt5, which may be direct or via RNA pol II.

Since c-Myc increases RNA pol II Ser-5 phosphorylation, which recruits CE and activates the guanylyltransferase, we investigated whether c-Myc increases CE activity [29, 35]. CE hydrolyses GTP to produce the GMP donor for the cap guanylylation reaction. Formation of the CE-GMP intermediate is used as an approximation of guanylyltransferase activity (Figure 1E) [35]. CE was immunoprecipitated from IMEC/c-Myc or IMEC/vec nuclear extracts and [α-^32^P]GTP supplied as the substrate, resulting in formation of a CE-[^32^P]GMP intermediate which was resolved by SDS-PAGE and quantified by phosphorimager (Figure 1F and 1G). Elevated c-Myc expression did not detectably alter total nuclear CE-GMP. However, only a fraction of CE is RNA pol II-bound and since it is this which catalyses capping, RNA pol II-associated guanylyltransferase activity was analysed. RNA pol II was immunoprecipitated from IMEC/c-Myc and IMEC/vec extracts and co-purifying CE-GMP complexes detected (Figure 1H). Elevated expression of c-Myc caused an increase in CE-GMP associated with RNA pol II, indicating that the CE recruited is active. In summary, c-Myc increases the recruitment and activation of CE.

### c-Myc promotes CE recruitment to c-Myc target genes

**Figure 2:**
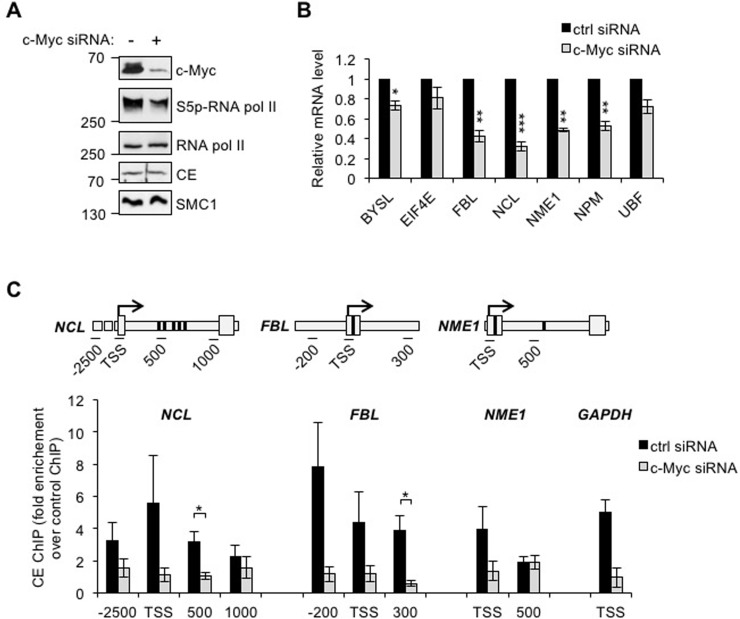
c-Myc regulates CE recruitment to c-Myc target genes (**A**) HeLa cells transfected with c-Myc siRNA or a non-targeting control for 24 hours were analysed by Western blotting. (**B**) c-Myc target genes transcript level, normalised to GAPDH, were determined by RT-qPCR in HeLa cells transfected with c-Myc siRNA or non-targeting control for 48-72 hours. Error bars represent standard error of the mean, n≥3. (**C**) Chromatin immunoprecipitation assay (ChIP) was performed using anti-CE antibody or control, in HeLa cells transfected with c-Myc siRNA or non-targeting control for 24 hours. c-Myc target genes (NCL, FBL and NME1), and GAPDH in ChIPs were analysed by qPCR. CE ChIP signal was expressed as fold enrichment over control ChIP signal. Schematics depict regions of primer amplification relative to the TSS (arrow) and E-box sequence(s) (bold band). Error bars represent standard error of the mean, n≥3. Significance calculated by Student's T-test; * = p≤0.05, ** = p≤0.01, *** = p≤0.001.

Since c-Myc increases the interaction of RNA pol II and CE, we investigated whether increasing c-Myc expression increases CE recruitment to target genes. CE recruitment was investigated in IMECs using chromatin immunoprecipitation (ChIP), however CE binding was below the limit of detection. As an alternative CE recruitment to c-Myc target genes was investigated in HeLa cells, which express high levels of deregulated c-Myc. In order to identify c-Myc target genes, Hela cells were transfected with c-Myc siRNA, which resulted in a decrease in c-Myc and phospho-Ser-5 RNA pol II (Figure 2A). A selection of target genes was analysed by RT-qPCR to identify the most c-Myc-responsive (Figure 2B). Nucleolin (NCL), fibrillarin (FBL) and nucleoside diphosphate kinase A (NME1) transcripts were the most significantly reduced in response to c-Myc suppression and thus the recruitment of CE to these genes was analysed by ChIP. CE binding to regions surrounding the transcription start site (TSS) and c-Myc-binding sites was analysed by qPCR (Figure 2C). Binding of CE to GAPDH TSS was also analysed. CE was found at the TSS of c-Myc target genes and proximally upstream. c-Myc depletion resulted in a decrease in CE binding to NCL, FBL, NME1 and GAPDH. Distal regions of NCL and NME1 served as a negative control for the ChIP. CE recruitment to these distal sites was low (~2-fold over background) and not c-Myc-responsive.

### c-Myc expression is dependent on CE

**Figure 3:**
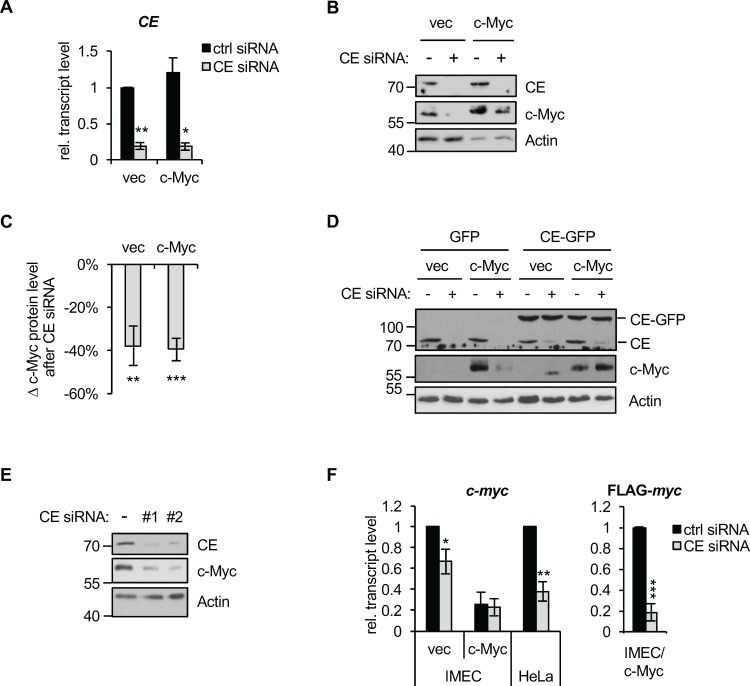
c-Myc expression is CE-dependent (**A**) IMEC/vec and IMEC/c-Myc were transfected with CE siRNA or non-targeting control for 72hrs. CE transcript expression normalised to GAPDH was determined by RT-qPCR. Error bars represent standard error of the mean, n=3 (**B**) IMEC/vec and IMEC/c-Myc transfected with CE siRNA (+) or non-targeting control (−) for 72hrs. Protein was analysed by Western blotting. (**C**) Densitometry was performed to quantify the decrease in c-Myc Western blot signal (normalised to actin) following CE depletion, relative to control transfection. Error bars represent standard error of the mean, n=6. (**D**) IMEC/vec and IMEC/c-Myc were stably transduced with GFP or a CE-GFP construct containing wobble codons to confer resistance to CE siRNA. Cells were transfected with CE siRNA (+) or control (−) and protein analysed by Western blot. (**E**) HeLa cells were transfected with two independent CE siRNAs (#1 or #2) or non-targeting control (−) for 72hrs. Protein analysed by Western blotting. (**F**) IMEC/vec, IMEC/c-Myc and HeLa cells were transfected with CE siRNA or non-targeting control. Endogenous c-Myc and Flag-c-Myc transcript level normalised to GAPDH was determined by RT-qPCR. Error bars represent standard error of the mean, n≥6. Significance relative to control siRNA calculated by Student's T-test, *** = p≤0.001; ** = p≤0.01; * = p≤0.05.

Since c-Myc regulates the recruitment of CE to its target genes, we investigated the dependency of their expression on CE. IMEC/c-Myc and IMEC/vec were transfected with CE siRNA resulting in CE transcripts and protein being depleted (Figure 3A and 3B). CE depletion resulted in a substantial reduction in the expression of endogenous c-Myc in IMEC/vec and exogenous c-Myc in IMEC/c-Myc (Figure 3B and 3C), which was rescued by expressing siRNA-resistant CE-GFP (Figure 3D). Reduced c-Myc expression was also observed in HeLa cells in response to transfection of two independent CE siRNAs (Figure 3E). Depletion of CE caused a reduction in endogenous c-Myc transcript levels in IMEC/vec and HeLa cells, and a reduction in exogenous c-Myc (FLAG-Myc) in IMEC/c-Myc. (Figure 3F). This suggests that reducing capping impacts the transcription or stability of c-Myc transcripts. Since exogenous c-Myc in IMECs is expressed without its endogenous promoter and untranslated regions, mechanisms involving the coding region determinant of c-Myc transcript stability may render it sensitive to degradation upon CE depletion [43, 44].

### Deregulation of c-Myc increases target gene dependency on CE

**Figure 4:**
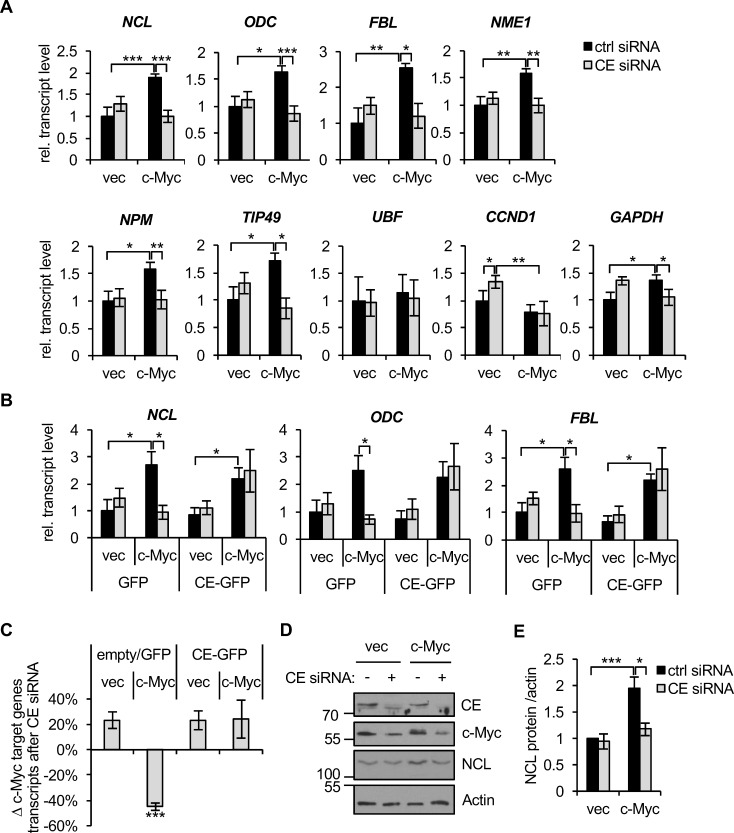
c-Myc-induced gene expression is CE-dependent (**A**) IMEC/vec and IMEC/c-Myc were transfected with CE siRNA or non-targeting control for 72hrs. c-Myc target gene transcripts were analysed by RT-qPCR, performed with equivalent amounts of RNA. Error bars represent standard error of the mean, n≥3. (**B**) IMEC/vec and IMEC/c-Myc expressing either GFP or CE-GFP (resistant to CE siRNA) were transfected with CE siRNA or control and transcripts analysed by RT-qPCR. Error bars represent standard error of the mean, n=4. (**C**) Average change in expression of c-Myc target genes (NCL, ODC, FBL, NME1, NPM and TIP49) upon CE depletion relative to control transfections. Error bars represent standard error of the mean, n=6. (**D**) IMEC/vec and IMEC/c-Myc were transfected with CE siRNA or non-targeting control for 72hrs. Protein analysed by Western blotting. (**E**) Densitometry performed to quantify the nucleolin (NCL) Western blot signal (normalised to actin) following CE depletion in IMEC/vec and IMEC/c-Myc. Error bars represent standard error of the mean, n≥3. Significance calculated by Student's T-test, *** = p≤0.001; ** = p≤0.01; * = p≤0.05.

The dependency of c-Myc target gene expression on CE was investigated using CE siRNA. Repressing CE expression will inhibit CE as a mediator of c-Myc function and as a controller of c-Myc expression, and have additional effects on gene expression. IMEC/c-Myc and IMEC/vec were transfected with CE siRNA and c-Myc target genes expression analysed by RT-qPCR (Figure 4A). NCL, ODC, FBL, NME1, NPM, FBL and TIP49 were verified as c-Myc-induced genes in IMECs. For these verified c-Myc target genes, the increase in expression induced by deregulated c-Myc was abolished by CE depletion. However, c-Myc target genes were unaffected by CE depletion in IMEC/vec, which only express endogenous *c-myc*. This was surprising given that endogenous c-Myc expression is impaired by CE knockdown and multiple independent experiments showed that CE levels are equivalently depleted in IMEC/vec and IMEC/c-Myc (Figure 3B and 3C). Expression of siRNA-resistant CE-GFP rescued the defect in c-Myc target gene expression in IMEC/c-Myc observed in response to CE suppression (Figure 4B, three representative genes, and Figure 4C, average result for 6 genes). GAPDH exhibited similar responses to CE suppression as the canonical c-Myc targets, suggesting that the interplay of CE and c-Myc influences genes on a global scale, albeit to a lesser extent with non-canonical targets (Figure 4A). UBF (upstream binding factor), a previously described c-Myc target gene, was unresponsive to c-Myc and CE knockdown in IMECs, indicating that c-Myc and CE dependency has a degree of specificity (Figure 4A). CCND1 (cyclin D1) is a c-Myc-repressed gene in IMECs [45, 46]. CCND1 transcript levels slightly increased when CE was depleted in IMEC/vec, correlating with reduced c-Myc protein (Figure 4A). However, when CCND1 was repressed by elevated c-Myc expression, it was unresponsive to CE depletion, suggesting that sufficient c-Myc remains to maintain CCND1 repression. Western blots were performed to determine if CE depletion affected the protein level of c-Myc targets in IMECs (Figure 4D and 4E). Consistent with transcript levels, nucleolin protein expression was impaired by CE siRNA in IMEC/c-Myc, but not in IMEC/vec.

**Figure 5:**
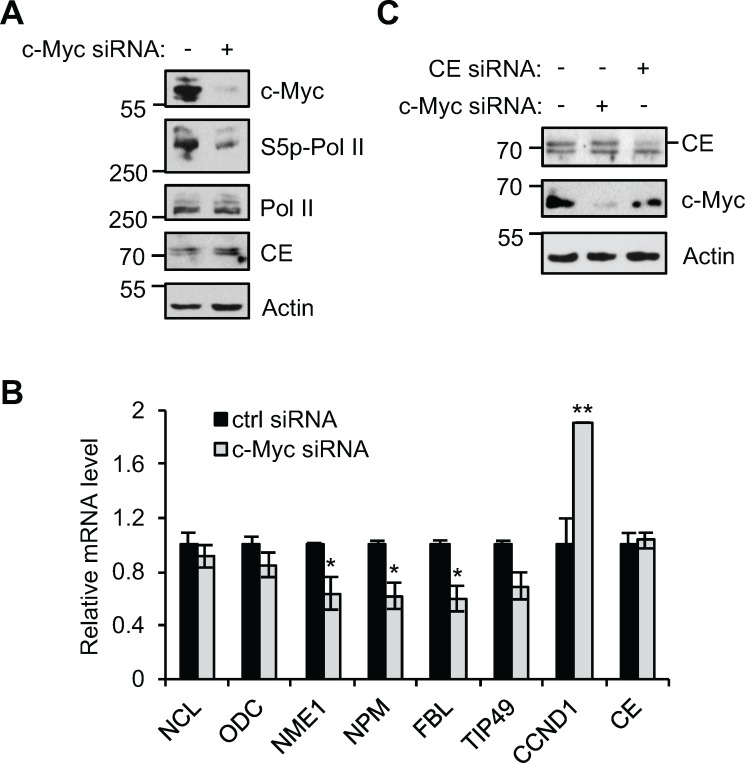
c-Myc target gene regulation in IMEC (**A**) IMEC/vec were transfected with c-Myc siRNA or non-targeting control for 72hrs. Protein analysed by Western blotting. (**B**) The expression of c-Myc target gene transcripts analysed by RT-qPCR, relative to GAPDH. (**C**) IMEC/vec were transfected with non-targeting control, c-Myc siRNA or CE siRNA for 72hrs. Protein analysed by Western blotting. Significance calculated relative to control siRNA using Student's T-test, ** = p≤0.01; * = p≤0.05.

We investigated why target gene expression was unaffected when endogenous c-Myc levels were reduced in response to CE depletion. It was possible that the c-Myc target genes analysed are only induced by deregulated and not endogenous c-Myc in IMECs. To investigate this, IMECs were transfected with c-Myc siRNA and knockdown of endogenous c-Myc confirmed (Figure 5A). RNA pol II Ser-5 phosphorylation decreased in response to c-Myc suppression, indicating that endogenous c-Myc is functional in IMECs. NCL, ODC and TIP49 transcripts were insensitive to this level of endogenous c-Myc knockdown, whereas NME1, NPM and FBL transcript levels were sensitive (Figure 5B). c-Myc siRNA caused a greater decrease in c-Myc than CE siRNA (Figure 5C). Therefore in IMEC/vec, NME1, NPM and FBL are target genes of endogenous c-Myc, but are resistant to the intermediate level of c-Myc depletion observed upon CE knockdown.

### Suppression of deregulated c-Myc reduces target gene dependency on CE

**Figure 6:**
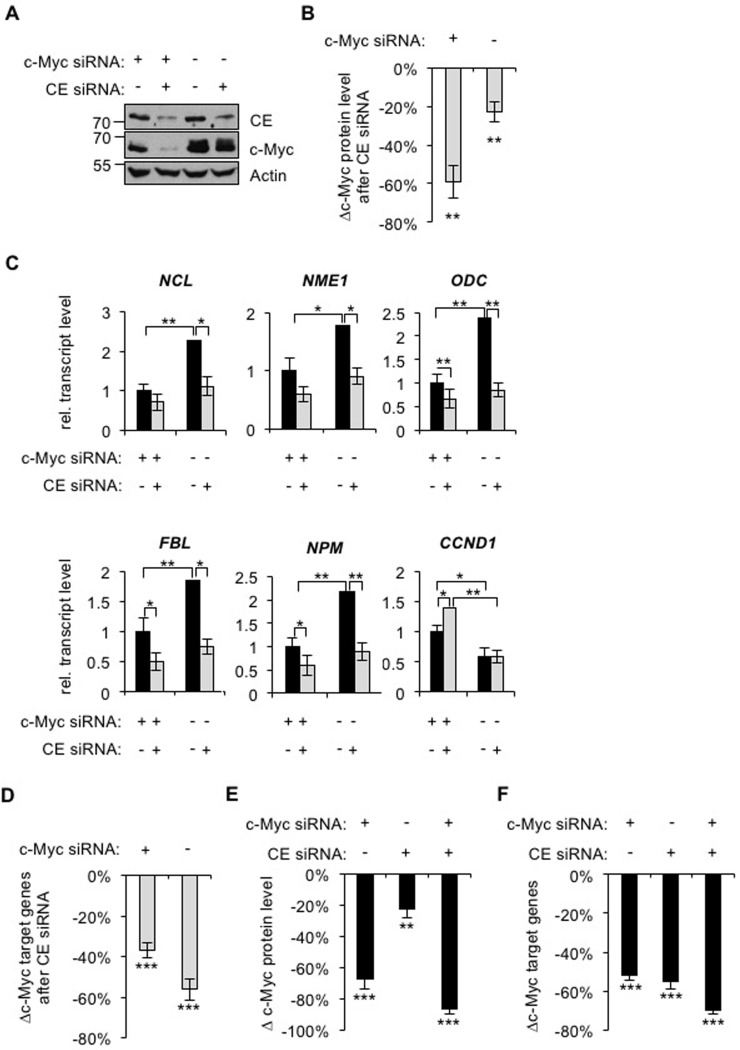
c-Myc depletion reduces target genes dependency on CE (**A**) HeLa cells were transfected with CE siRNA, c-Myc siRNA and/or non-targeting control for 72hrs. Protein analysed by Western blotting. (**B**) Densitometry performed to quantify the decrease in c-Myc Western blot signal (normalised to actin), in response to CE depletion in HeLa cells, transfected with c-Myc siRNA or non-targeting control. Error bars represent standard error of the mean, n≥6. (**C**) c-Myc siRNA, CE siRNA and control transfections were performed for 72 hours. c-Myc target gene transcripts were analysed by RT-qPCR, normalised to GAPDH. Error bars represent standard error of the mean, n=4. (**D**) Average change in c-Myc target gene transcripts (NCL, NME1, ODC, FBL and NPM) in response to CE siRNA, relative to control transfections. Error bars represent standard error of the mean, n=5. (**E**) Decrease in c-Myc Western blot signal following c-Myc and/or CE knockdown, relative to control transfection. Error bars represent standard error of the mean, n≥6. (**F**) Average decrease in c-Myc target gene transcripts (NCL, NME1, ODC, FBL and NPM) following c-Myc and/or CE knockdown, relative to control transfection. Error bars represent standard error of the mean, n=5. Significance was calculated by Student's T-test, ***= p≤0.001; **= p≤0.01; *= p≤0.05. For B and D-F, significance relative to control siRNA.

We investigated if c-Myc target gene expression is dependent on CE in HeLa cells, which have deregulated transcriptional activation of the *c-myc* gene due to viral insertion [47]. Cells were transfected with combinations of c-Myc and CE siRNAs or non-targeting controls, which resulted in efficient knockdown of both proteins (Figure 6A). c-Myc depletion was detected 24 hours after c-Myc-siRNA transfection whereas CE depletion was not detectable until 48 hours after CE siRNA transfection (data not shown). Therefore, in this experimental arrangement CE is depleted following changes in c-Myc protein level. As in IMECs, c-Myc expression was dependent on CE regardless of c-Myc levels, although this was more apparent in when c-Myc was repressed by transfection of c-Myc siRNA (Figure 6A and 6B). CE depletion in HeLa cells resulted in significant repression of c-Myc target genes (Figure 6C). When c-Myc was repressed, CE depletion resulted in reduced expression of some c-Myc target genes, but to a lesser extent than that in HeLa control cells which carry deregulated c-Myc (Figure 6C, specific genes; Figure 6D, average result for five genes). This indicates that deregulated c-Myc in HeLa cells causes enhanced dependency of c-Myc target genes on CE. As in IMECs, the c-Myc-repressed gene CCND1 was unaffected by CE depletion in cells expressing high levels of c-Myc.

We questioned whether CE knockdown only influences c-Myc target gene expression by regulating c-Myc. Transfection of CE siRNA in HeLa cells caused, on average, a ~20% reduction in c-Myc expression whereas c-Myc siRNA caused a 70% reduction (Figure 6E). However, transfection of CE siRNA depletes c-Myc target gene expression to the same extent as transfection of c-Myc siRNA (Figure 6F, average decrease in expression of five genes). This suggests that CE does not control c-Myc target genes simply by regulating c-Myc expression; its role as a mediator of c-Myc function is also important. In addition, we note that other genes are likely to be sensitive to CE depletion which may also impact on c-Myc target genes.

### c-Myc-driven cell proliferation and transformation is dependent on CE

**Figure 7:**
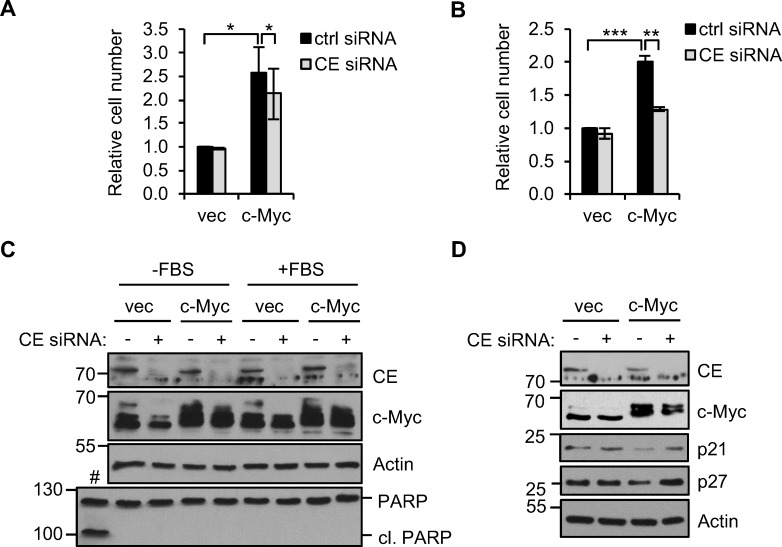
CE depletion reduces c-Myc-dependent cell proliferation. (**A**) IMEC/vec and IMEC/c-Myc were transfected with CE siRNA or a non-targeting control and counted after 72 hours. Error bars represent standard error of the mean, n=3. (**B**) IMEC/vec and IMEC/c-Myc were maintained in growth medium supplemented with 5% FBS. Cells were transfected with CE siRNA or control and counted after 72hrs. Error bars represent standard error of the mean, n=3. (**C**) IMEC/vec and IMEC/c-Myc cultured with or without FBS were transfected with CE siRNA or control for 72hrs. Protein analysed by Western blotting. IMEC/c-Myc treated with 10μM MG123 for 24 hours as a positive control for apoptosis (#). (**D**) IMEC/vec and IMEC/c-Myc were transfected with CE siRNA or control and protein analysed by Western blot. Significance calculated by Student's T-test, *** = p≤0.001; ** = p≤0.01; * = p≤0.05.

In epithelial cells, deregulated c-Myc increases cell proliferation and induces anchorage-independent cell growth, characteristics of many epithelial cancer cells. Since deregulation of c-Myc increases target gene dependency on CE, we investigated whether cell proliferation and anchorage-independent growth is also CE-dependent. Consistent with previous studies, deregulated c-Myc in IMECs increased cell proliferation (Figure 7A and 7B) [38]. IMEC/c-Myc and IMEC/vec were transfected with CE siRNA and cells counted after 72 hours. IMEC/vec proliferation was unaffected by CE depletion, however, IMEC/c-Myc cell number was significantly reduced.

To determine whether CE depletion influences c-Myc-driven cell transformation, we performed anchorage-independent cell growth assays. The IMEC culture medium is serum-free, however anchorage-independent growth assays require a supplement of fetal bovine serum (FBS) [45,48]. c-Myc increases the proliferation of IMECs to a similar extent in the presence or absence of FBS (Figure 7A and 7B). c-Myc expression was not altered by culturing cells in FBS (Figure 7C). In a previous study, CE depletion induced apoptosis in HeLa cells [49]. CE siRNA did not induce PARP cleavage in IMECs or morphological signs of apoptosis (Figure 7C, data not shown). The p21 and p27 tumour suppressor genes are c-Myc-repressed genes which negatively regulate the G1-S transition in the cell cycle [50]. p21 and p27 were elevated by CE depletion in IMEC/c-Myc but not IMEC/vec (Figure 7D). This indicates that CE knockdown restrains c-Myc-driven cell cycle transit in IMECs.

**Figure 8:**
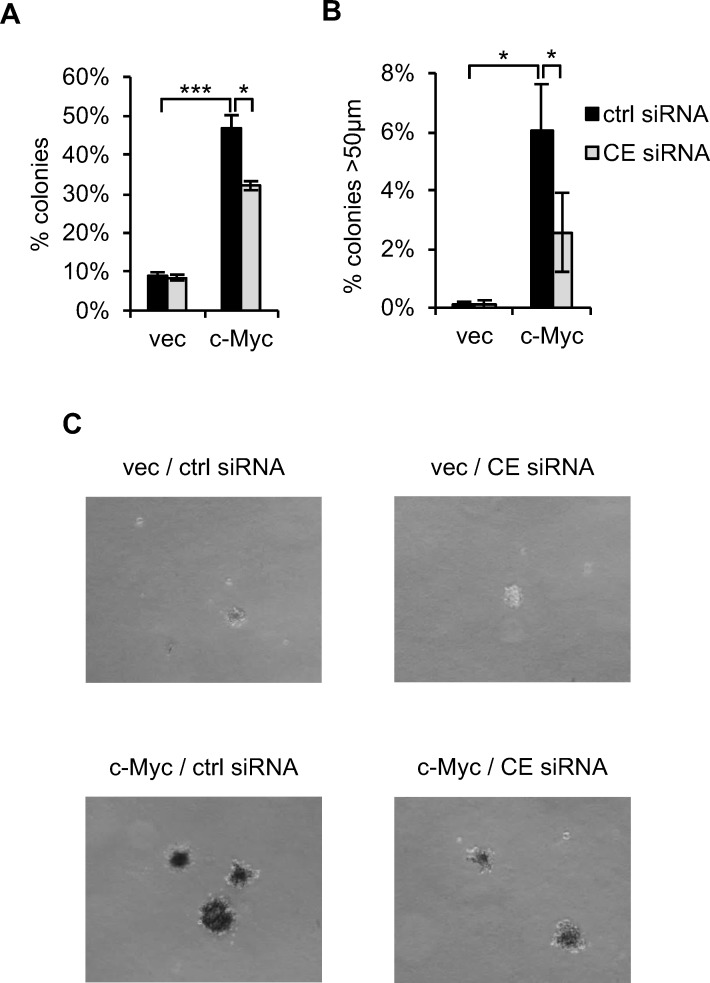
CE is required for c-Myc-induced transformation (**A**) IMEC/vec and IMEC/c-Myc maintained with 5% FBS were transfected with CE siRNA or a non-targeting control. After 72 hours cells were plated in suspension. Colonies scored using a graticule after 9-15 days. The percentage of cells forming colonies >20μm reported. Error bars represent standard error of the mean, n=3. (**B**) The percentage of cells forming colonies >50μm reported. Error bars represent standard error of the mean, n=3. (**C**) Micrographs taken one month after cells were plated in suspension. Scale bar represents 200μm. Significance calculated by Student's T-test, ***p≤0.001; *p≤0.05.

IMEC/c-Myc and IMEC/vec were transfected with CE siRNA prior to the soft agar transformation assays which measure anchorage-independent cell proliferation. Deregulated c-Myc expression resulted in 45% IMECs plated growing in anchorage-independent colonies (Figure 8A) [45,51]. CE depletion significantly reduced anchorage-independent colonies, including reducing the proportion of colonies over 50μm in diameter by 60% (Figure 8B and 8C). Therefore, CE is important for the incidence of c-Myc-dependent transformation and for subsequent colony growth.

## DISCUSSION

c-Myc is a potent oncogene and one of the most frequently deregulated in human cancers. The majority of selective cancer therapies function by targeting the active site of key enzymes with small molecule inhibitors. However, c-Myc has been a challenging target since it lacks an active site and as a result there has been sustained interest in its mode of action, particularly in its enzymatic co-factors. In this study we investigated the relationship between c-Myc and the mRNA capping enzyme CE/RNGTT, which initiates mRNA cap formation by adding an inverted guanosine group to nascent transcripts. The mRNA cap protects the transcript from degradation during transcription and recruits processing and translation factors. We determined that deregulated c-Myc increases the interaction of CE and RNA pol II, increases CE recruitment to c-Myc target genes and increases the CE guanylyltransferase activity associated with RNA pol II. Furthermore, we observed that CE is important for c-Myc expression. Deregulation of c-myc co-ordinately promotes many major metabolic pathways in the cell resulting in enhanced cell proliferation and proliferation becoming somewhat independent of growth controls. In IMECs and HeLas, cell proliferation and transformation driven by deregulated c-Myc were found to be CE-dependent.

Cells carrying deregulated c-Myc were significantly more dependent on CE for gene expression, cell proliferation and cell transformation than cells with low c-Myc expression. IMECs which express low endogenous c-Myc levels or HeLa cells in which c-Myc expression was supressed by siRNA were largely unaffected by transfection of CE siRNA. This was somewhat surprising given that endogenous and deregulated c-Myc expression is dependent on CE. Deregulated c-Myc results in a substantial increase in global transcription which may result in increased dependency on CE to maintain transcript processing. Several previous studies in cancer cells have shown that c-Myc expression thresholds govern distinct cellular responses [52–54]. The molecular mechanisms governing these c-Myc thresholds are likely to involve the availability of transcriptional co-factors and repressors.

Since the discovery that c-Myc regulates mRNA capping, studies have focussed on the involvement of RNMT, the N-7 guanosine cap methyltransferase, which completes the basic functional cap structure [8, 55]. Mechanisms involving RNMT recruitment and methionine metabolism contribute to c-Myc-dependent cap methylation [8,27]. Inhibition of RNMT function by blocking biproduct removal selectively targets cells carrying deregulated c-Myc. The discovery that c-Myc regulates recruitment of CE reveals that c-Myc is co-ordinating all the basic mechanisms of mRNA cap formation. Inhibition of both guanosine cap addition and guanosine cap methylation selectively target cells with deregulated c-Myc, indicating that both steps in cap formation are critical for c-Myc function [8, 27].

Transcription and translation are considered attractive therapeutic avenues for targeting c-Myc-driven cancers, since c-Myc deregulation increases global transcript and protein production [23, 25, 56–59]. Recent studies reported that many cancer cell lines are sensitive to Cdk7 inhibitors, which reduce RNA pol II Ser-5 phosphorylation [60, 61]. The CDK7 inhibitor THZ1 is highly effective in targeting models of c-Myc-driven small cell lung carcinoma, neuroblastoma and triple-negative breast cancer [62–64]. Among its functions, THZ1 abolishes co-transcriptional mRNA capping, which may contribute to its efficacy [65]. Other components of the gene expression machinery are being investigated as therapeutic targets in c-Myc-driven cancers, including the splicing machinery and cap binding proteins. Components of the splicing machinery are direct targets of c-Myc and are essential in c-Myc-driven lymphamogenesis [66]. Other splicing components are synthetic lethal with overexpressed c-Myc [67]. Inhibiting the function and interactions of the translation initiation complex, eIF4F, interferes with cap-dependent translation and is also being investigated as a therapeutic strategy [68]. The data presented here demonstrate that deregulated c-Myc sensitises cells to inhibition of the Capping Enzyme, CE. Inhibiting CE targets c-Myc expression and function and therefore we suggest that CE should be investigated further as a therapeutic target.

## MATERIALS AND METHODS

### Cell culture and manipulation

Cells maintained at 37°C/5% CO_2_ in humidified incubator. IMECs cultured according to [37], or supplemented with 5% FBS (see figure legends). HeLa cells cultured in Dulbecco's modified eagle medium (DMEM)/10% FBS/2mM L-glutamine. PhoeNX cells cultured in DMEM/10% FBS/2mM L-glutamine/1mM sodium pyruvate. Cells counted in 0.2% trypan blue using Countess cell counter (Life technologies). For retroviral infection, 10cm plate PhoeNX cells transfected with 4ug DNA using 8ug polyethylenimine. After 48hrs, viral supernatant 0.45μm filtered, mixed 1:1 with cell media and 5μg/ml polybrene and added to recipient cells. After 24-72 hours cells selected with 150μg/ml hygromycin B (LXSH c-Myc constructs) or 500μg/ml G418 (pBMN-IRES-Neo CE-GFP constructs). siRNA transfections performed using Lipofectamine RNAiMAX (Life Technologies). Dharmacon siRNAs used (non-targeting control: D-001210-03-50, c-Myc: D-003282-14-0050, CE #1: D-009782-01-0050 and CE #2: D-009782-02-0050). Cells transfected during seeding using 50nM siRNA or 75nM for double target knockdowns.

### Cell extracts

Performed on ice/4°C. Cells lysed in F buffer (10mM Tris/Cl pH 7.5, 50mM NaCl, 30mM Na_4_ pyrophosphate, 50mM NaF, 5μM ZnCl_2_, 10% glycerol, 0.5% Triton x-100) supplemented with 1mM DTT, 1μM pepstatin, 10μM leupeptin, 0.1 trypsin inhibitor units aprotinin, 1% phosphatase inhibitor cocktail 2 and 3 (all Sigma Aldrich). Protein concentration determined by Bradford assay. For nuclear extracts, cells swelled in Buffer A (10mM HEPES pH 7.9, 1.5mM MgCl_2_, 10mM KCl) and lysed by syringing with 27G needle. Nuclei centrifuged at 6000rpm for 10 minutes and lysed in F buffer.

### Mass spectrometry

Performed by LC-MS-MS using linear ion trap-orbitrap hybrid mass spectrometer (Orbitrap-Classic, Thermo) equipped with nanoelectrospray ion source (Thermo) and coupled to a Proxeon EASY-nLC system. Peptides prepared by in-gel tryptic cleavage were injected via 2cm trap column (Nano Separations, NS-MP-10 BioSphere C18, 5μm, 120Å,360/100μm) onto Thermo (Part No. 160321) Acclaim PepMap100 reverse phase C183μm column, 75μm x 15cm, with 300 nl/min flow and eluted with linear gradient of 95% solvent A (2% Acetonitrile/0.1% formic acid/H_2_O) to 35% solvent B (90% acetonitrile/0.08% formic acid/H_2_O) at 20 minutes followed by rise to 80% B at 23 minutes, maintained at 80% B for 5 minutes, followed by re-equilibration. Instrument operated with ‘lock mass’ option to improve mass accuracy of precursor ions and data acquired in data-dependent mode, automatically switching between MS and MS-MS acquisition. Full scan spectra (m/z 350-2000) acquired in orbitrap with resolution R=60,000 at m/z 400 (after accumulation to FTMS Full AGC Target; 1,000,000; MSn AGC Target; 100,000). 5 most intense ions, above specified minimum signal threshold (5,000), based upon low resolution (R = 15,000) preview of the survey scan, fragmented by collision induced dissociation and recorded in linear ion trap, (Full AGC Target; 30,000. MSn AGC Target; 5,000). Proteins were identified and quantified using Maxquant software. RNA pol II mascot scores 3545 and 2529.

### Western blotting

Primary antibodies used: CE (in house); c-Myc (9402), Spt5 (9033) and PARP (9542) from Cell Signalling; tubulin (sc-9104), RNA pol II pan (sc-899) and p21 (sc-397) from Santa Cruz; RNA pol II S5-P CTD (3E8) from Chromotek; actin (ab3280) and p27 (ab32034) from Abcam; and SMC1 (A300-055A) and nucleolin (A300-711A) from Bethyl Laboratories.

### Immunoprecipitation

Immunoprecipitations (IPs) performed at 4°C in F buffer. CE-GFP co-IPs performed for 2.5 hours using GFP-Trap_A (Chromotek). CE co-IPs performed for 2.5 hours with 2μg CE antibody (in house). For guanylyltransferase assays, CE IPs performed for 2.5 hours with 0.5μg CE antibody and RNA pol II IPs performed overnight with 4μg Poll II (pan) antibody (Santa Cruz, sc-899). For endogenous CE and Pol II IPs, 20μl Protein G agarose bead slurry (Generon) added for 1 hour. IPs washed three times in 10mM Tris/Cl pH 7.5/150mM NaCl/0.5mM EDTA and eluted in F buffer/Laemmli buffer/0.1M DTT. Samples analysed by SDS-PAGE.

### Chromatin immunoprecipitation

Chromatin immunoprecipitation (ChIP) performed as described [69]. Two 10cm plates of subconfluent cells used per ChIP. Cells crosslinked for 15 minutes. 1μg polyclonal CE antibody used per ChIP. 1μl (2% total) ChIP DNA or diluted input DNA used per 10μl quantitative PCR (SsoFast EvaGreen supermix, Bio-Rad). 3-fold input DNA dilutions used to calculate primer efficiency.

### Reverse transcriptase PCR

RNA extracted using TRIzol (Life Technologies). cDNA synthesis reactions (20μl) containing 200ng RNA prepared using iScript cDNA synthesis kit (Bio-Rad). 0.5ul cDNA per 5μl qPCR (SsoFast EvaGreen supermix, Bio-Rad). 3-fold cDNA dilutions used to calculate primer efficiency.

### Guanylyltransferase activity assay

Guanylyltransferase activity assays performed as described [31]. Cell lysis and IPs performed in F buffer/5mM DTT. Washed IPs incubated with 3.75U/ml RNase A/150 U/ml RNase T1 for 15 minutes at 20°C. IPs washed three times in reaction buffer (50mM Tris pH 7.8/5mM MgCl2/5mM DTT), then incubated for 1 minute at 37°C in reaction buffer/6.7μM [α-32P]GTP (Perkin Elmer, BLU006H500UC). IPs analysed by SDS-PAGE. Gels dried and CE-GMP intermediate quantified by phosphorimager.

### Anchorage-independent cell growth assay

8,000 cells plated in 2ml growth medium/5% FBS/0.33% noble agar in 6 well plate on top of 0.6% noble agar base layer (2ml). Technical triplicates performed. Cells fed with 500μl growth medium/5% FBS alternate days. After 9-15 days, colonies in five fields from each well scored using graticule.
